# New Jersey State Dental Society

**Published:** 1883-09

**Authors:** 


### NEW JERSEY STATE DENTAL SOCIETY.

The proceedings reached us in such a fragmentary form that we have been obliged to omit some parts because of a loss of connection, and to supply others.—Editor.

The thirteenth annual meeting of this Association was held at the Coleman House, Asbury Park, N. J., commencing July 18, 1883. The President was Dr. J. G. Palmer, of New Brunswick, and the Secretary, Dr. Chas. A Meeker, of Newark.

The President delivered an excellent annual address, which, however, was chiefly local in its application, and after the usual routine business, Dr. C. S. Stockton said :

Mr. President and Gentlemen,—You are all more or less familiar with the journal called The Independent Practitioner, which has been a joining together of dental and medical affairs, and has been a representative, more or less, of dental instrument makers and dental goods houses. I wish to call your attention to the fact that this Independent Practitioner will in the future be edited by Dr. W. C. Barrett, of Buffalo, and that it will be under the management of a number of gentlemen in New York, who are entirely independent of dental houses and dental goods dealers. We are under obligations, I know, to dental houses for the manner in which they have favored and assisted dental societies, and it may be that we would never have attained the position we have without their assistance, but the time has arrived when we want an Independent Dental Journal, and I can assure you from my close personal acquaintance with Dr. Barrett, and my knowledge of the gentlemen who are associated with him, or connected with this journal, that we will have in The Independent Practitioner all that we desire in an independent journal. I therefore take pleasure in calling your attention to it, and I hope every member of this Society will subscribe for it, in order to encourage this new departure in independent journalism, as well as for their own benefit. I have a number of copies here for free distribution. I have a *carte blanche* to receive subscriptions, and if any gentleman wishes to subscribe, after he has inspected a copy, I shall be very glad to receive his name. Furthermore, I will state that the proceedings of this Society will be published almost entire in this Journal, and it becomes us, if we want a copy of those proceedings, to subscribe for the journal that has the enterprise to print them.

The President—I desire to add a matter of my own knowledge to what Dr. Stockton has said, which is that hereafter the proceedings of the regular monthly meetings of the Central Dental Association of Northern New Jersey, will be published in THE INDEPENDENT PRACTITIONER, and that may possibly make the journal more desirable to the members of this Society, at least.

Dr. Henry S. Chase, of St. Louis, not being present to read his paper on Filling Teeth, it was read by Dr. C. W. Meloney.

This paper has not been received—EDITOR.

Dr. J. Hayhurst, of Lambertville, read a paper entitled Filling Teeth and the Philosophy Thereof. (See page 465.)

*Dr. Bodecker, of New York*—Mr. President and Gentlemen—In the paper of Dr. Chase we again have the electrical theory of dental caries advanced. This theory, I believe, has very little ground; in fact it is a settled question that the dentine as well as the enamel are classed among the worst conductors of electricity. If there are any manifestations of electrical action in the dentine, it is due to the moisture contained in the canaliculi. Dr. Willoughby Miller, of Berlin, who may be regarded an authority in dental caries, has made a series of very valuable experiments in the laboratory of Prof. E. du Bois Reymond, of Berlin, (see INDEPENDENT PRACTITIONER for February, 1883,) wherein he has arrived at the conclusion that a piece of dentine or enamel, when dry, subjected to the test of a very delicate galvanometer, scarcely showed any deviation of the needle; but if, on the other hand, the dentine was moistened with a physiological solution of chloride of sodium, a deviation was observable. Dr. Miller found that no galvanic action exists between the tooth itself and the filling material; but if different metallic fillings were placed in the same mouth, a galvanic current from the more oxydizable (the electro positive) to the less oxydizable (the electro negative) metal, is the result, as long as the surfaces of these metals remain unoxydized. This oxydation in the positive metal, (amalgam or tin) fortunately does occur, and with it the electrical current gradually is destroyed. Dr. Miller furthermore found that on the surface of every filling, and especially of gold if unevenly condensed, an electric current can be detected as long as the surface remains clean. If this is the case, a gold filling introduced by the aid of the electro-magnetic mallet would be the best that can be made with regard to electrical action, for I believe with no other mallet can we introduce gold so uniformly as with this.

Dr. Chase furthermore says, if I understand him correctly, that gold is the worst material to fill teeth with, and a permanent filling can not be made with it. The Doctor may be right if he speaks of very badly organized teeth, and when we consider the process of secondary decay from a rational standpoint we will understand why most of the gold fillings in this class of teeth do not stand. Secondary decay, I believe, is nothing but a low grade of inflammation, mostly due to the irritation of the metallic filling. Gold, when in direct contact with the dentinal fibers, acts as a foreign body, and thus produces what is termed, "eburnitis;" that is, the Tomes fibres swell, the surrounding lime salts are dissolved, and the basis-substance breaks down, and in microscopical specimens we will find that the carmine (if stained) has been taken up by the Tomes fibres very much more than in other parts of the same specimen. But in fairly organized teeth gold can be made a perfect filling every time, if you protect the living matter of the dentine by something like oxy-chloride or oxy-phosphate of zinc. With this precaution gold is a better filling material than any other substance. If a cavity is lined with a solution of gutta-percha, then partially filled with oxy-chloride or oxy-phosphate of zinc, and a gold filling put over that, I do not believe there will, except in rare instances, be any secondary decay, always provided the gold filling has been put in water-tight.

As for the imperfection of gold fillings, I do not think there are so many as is claimed. Most of them are inserted pretty well, and stand pretty well under ordinary conditions. When fillings give way the fault is more in the substance of the tooth, I think, if the edges of the filling were made perfect when inserted. We see in the mouths of some persons fillings that have been there for twenty, thirty, or forty years, many of which might be picked out with a nerve broach, and yet they remained all that time. In such cases it is decidedly not the workmanship that saved the teeth, but the density of the tooth-substance.

*Dr. Atkinson.*—Mr President and Brethren—I deem it an auspicious day when we can gather a hundred dentists together to talk upon subjects that have puzzled the wisest heads in physics and physiology,and have them speak with some little apprehension of what they are talking about; but when a man who has a large reputation, large experience and large imagination forgets the line between cause and effect, between antecedent and sequence so far as to charge to the material he works with the imperfections of his manipulative ability, it is time he was criticised so severely as to prevent the injury he would do to young men who may be within hearing of the paper to which we have listened. It is hardly possible to get as full a discussion of a question anywhere else as we have in our Dental Associations, for we are a young body, and are not overloaded with false precedent, with mere notions passing for scientific attainment.

I must commend Brother Hayhurst's paper; perhaps his Quaker blood explains that; but he should understand that the power is not the machine, but is that which operates the machine. If there is any analogy between a case of secondary decay in a tooth and the individuals out of whom the evil spirits were driven, it would not be attributable to the destruction of the evil spirits, but to the larger territory that the new comers would have to act upon, and he must understand that when we say spirit we do not mean an invisible figment, but a concentration of power in an awakened presence, holding within it a purpose. All this trouble about understanding what decay is has come from invariably attributing it to the seat of the activity rather than to the activity itself, and if it comes down to saying whether we really know what decay is, or that we do not know, it depends upon our definition of the term decay. If we follow the term back to its origin we find that it comes from the Latin *de* down, and *cado*, I fall, and then we do know what it is; it is a falling apart of the elements that have been built up, whether it be in the form of enamel, dentine, muscle, nerve, or connective tissue fibre. I rejoice that the day has come when we can see with something of the inspiration of John Bell, a prescience which they tried to laugh him out of, but which has become definitely proved by many individuals who have had the patience to examine the process of the breaking down of the dentine. There is an abscess of the dentine, a retrogressive metamorphosis without any immediate connection with the cavity of the mouth through broken down tissues, the enamel being entire, and this proves that the process is inflammatory. And that was seen as long ago as John Bell's time. He did not have the great advantages that Brother Bodecker has had and improved, under such fine instruction as is obtainable in Carl Heitzmann's laboratory. But now it is revealed to us that the destruction of tissue has to follow the same law that governed its building up, by a reversal of the action.

The seat of the inflammatory action of which Dr. Bodecker has spoken, is in the dentine, and that inflammatory action is nothing else than a return of the tissues to their embryonic condition.

One thing that Dr. Bodecker said may make you stumble. He said the dentinal fibrillæ swell. They cannot swell unless they have space to swell in. His statement is true, but not sufficiently complete. The lime salts are first dissolved, leaving the animal basis-substance, or proteinaceous matter, and then, from the greater activity of the nutrient action in the dentinal fibres, they encroach upon the basis-substance of the dentine, which is now in a proteinaceous condition. What then is the result? That which was considered a mineral body before has now become changed into a higher grade of nutrient activity, and holds an affinity for analine, for salts of nitrate of silver, etc., which are capable of coloring the living tissue, and by which the microscopist detects the difference between the organic and the inorganic substance.

Miller has worked well. He has shown that it is the water that is the conductor of this mode of motion that we call electricity, and not the dry dentine, or the dry enamel.

As long as the enamel is normal it has about two per cent of animal matter in it, and that contains hardly enough of the water element to induce much conduction ; but some very hyper-sensitive people, whose teeth probably have larger protoplasmic strings than those of better organization, will have the enamel of their teeth "set on edge" by taking acids into the mouth. How can we cure teeth that are so affected? By simply applying a little soap or soda. How is that done? You have not restored all the lime salts that the acid had removed. No, but you have restored that finer thing that is essential to normal sensibility, the harmony of the nutrient currents.

*Dr. Osmun.*—I feel somewhat embarrassed in taking the floor to say anything regarding these papers after such speakers as Dr. Atkinson and Dr. Bodecker have discussed them. But I think the paper of Dr. Chase is capable of doing a great deal of harm. I grant that he may be an earnest seeker after truth, and that he intends to do good; but I think that if there is anything that has been detrimental to the young members of the profession it is this everlasting cry of plastic fillings. When men like Professor Flagg, Dr. Chase and Dr. Palmer, of Syracuse, advocate such a system as that, the young men of the profession are very apt to follow in their footsteps and serve these plastic idols instead of their golden Gods; they are readily induced to take up the plastic fillings, because they are worked a little easier and more comfortably, and do not take quite so much time; hence, I think such papers are likely to do a great deal of harm. When I first read Dr. Foster Flagg's book I was very much interested in it; when I read Dr. Chase's articles I thought I had found something that would fill the bill; that would enable me to serve people quicker and more cheaply, and do a greater amount of good than I could with gold, which requires hours and hours of time to make a perfect operation. I saw in that plastic material something that would enable me to do a great deal more work and so serve a greater number of people. But, gentlemen, as I stand here to-day, I think it is one of the greatest curses that has ever invaded dentistry. I have gone back to the old way, and I tell you that gold foil manipulated with care, gold foil impacked with careful, conscientious manipulative ability, is the only filling which will stand by you through thick and thin. Is not that so, Dr. Atkinson?

*Dr. Atkinson.*—With one amendment. Gold is a conductor of thermal and electrical currents; but if you will put a little oxy-phosphate, or oxy-chloride of zinc, or gutta-percha over the dentine before you introduce your gold, then I say amen to every word.

*Dr. Osmun.*—I accept the amendment. And I protest in the name of every young dentist now in the profession or who shall come into it hereafter, against such teachings as that, for they are simply damnable.

*Dr. Watkins.*—Dr. Atkinson says you should partly fill the tooth with gutta-percha, and pack the gold over that. I did not know that he ever used gutta-percha under gold fillings. I have used it and have seen it used packed in solid with gold on top of it, and it split the tooth open. I have seen other cases where the side of a tooth so filled was broken off. I would like to hear something on that point.

*Dr. G. Carleton Brown.*—I remember hearing, a few years ago, at the Pennsylvania College, Dr. Truman, I think it was, say that he did not believe a tooth was ever saved that was capped with gutta-percha. He illustrated his idea with a great many cases, and many persons agreed with him. Since then there has been another revolution, I believe, and some have gone back to gutta-percha.

*Dr. Fowler.*—My experience is that a gold filling, well put in, is the filling of all fillings. I have used plastic fillings with good success in their place, but of all fillings gold, when properly inserted, is the best.

*Dr. Louis A. Reading.*—With reference to non-conductors under metallic fillings, I have had no experience except with phosphates. I have used them in such cases with wonderful success by making good anchorages, not only up near the free margin of the dentine, but also making very small retainers in the phosphates.

*Dr. J. A. Woodward.*—Some time ago my attention was drawn to some reported experiences in relation to the bursting or forcing away of portions of the crowns of teeth filled with gutta-percha, and covered with some unyielding material, and I was led to make the following experiment. Samples of three of the standard makes of gutta-percha were packed into six glass tubes. One filling of each make was then tested, and found to leak as usual. The other three tubes and fillings were placed in water at the ordinary temperature, for three days, and then tested, and were found not to leak. This result is due to the expansion of gutta-percha under moisture. When a large mass of gutta-percha is packed into a cavity and covered with any filling which will confine it, the expansion of the gutta-percha from heat and moisture undoubtedly exerts very considerable force in all directions. Should the tooth be a frail one, slight external force may cause a fracture; or should the pulp be closely encroached upon, pulp irritation may follow from the pressure.

*Dr. M. L. Rhein.*—Concerning the covering of the bottom of cavities with oxy-phosphate and then packing gold over it, I would say that I have followed that plan of procedure during the past three-and-a-half years, and I have not found a single case in which I had cause to regret it. My plan has been about the same as that described; that is, not to make retaining-points in the oxy-phosphate, but merely starting-points, and if any anchorage is needed other than the shape of the cavity affords, make it near the free margin of the dentine and enamel.

Besides that form of capping we find a large number of teeth where the cavities are such as not to permit us to place in them sufficient oxy-phosphate to afford the needed protection. This is especially the case with laterals, and they are the teeth which need a non-conducting barrier the most, they being the worst teeth for gold to be placed in immediate contact with the dentine. In those teeth I obtain the greatest benefit from the use of fine silk court-plaster. It can be cut in any desired shape, moistened and placed over the dentine, and the gold packed directly against this, forming a barrier which will prevent any thermal changes. I have found its employment successful in almost every case where I have used it; after the operation of restoring contour, etc., is completed, the application of cold or heat should not produce the slightest sensation.

I would like to touch upon another point in the discussion, and that is the use of plastic fillings. Every one who has preceded me has decried the use of amalgams, intimating that nothing could save the teeth but gold. I strongly believe that a tooth can be better preserved through the aid of gold, cohesive gold, properly packed, but we should not ride the hobby of gold or no filling at all. Let a few grains of common sense be added to that golden rule. A great many patients come to us with the teeth in the posterior portion of the mouth in a broken down, bad contour, and the patient will not and cannot spend either the time or the money necessary to have them restored to their natural contour with gold. Must such teeth eventually be consigned to the mercies of the forceps? Is conservative dentistry merely for the wealthy? I have found that the use of a proper kind of alloy, impacted in a proper manner, will save those teeth, and will not discolor the tooth-structure. The best filling material I have found for that purpose is Bonwill's gold and platinum alloy. The quality of material is one consideration, but a more important point is that the work must be done in as thorough a manner as with gold foil. In this connection remember to take up all the free mercury. The neglect of this point causes most of the failures. It is very difficult to mix any alloy without leaving some free mercury in it. Use the mallet in inserting the amalgam, and by placing tin foil under the mallet you take up all the free mercury. The utmost care should be observed to take up every particle of the excess at the margin of the filling and the enamel, and by using that precaution, no discoloration of the tooth structure will ensue. A good amalgam, worked properly, will save teeth. I do not think it preserves them as well as gold, and I think I echo the sentiment of a great many dentists when I say it is *easier* to insert a *perfect gold filling* than to insert a good *amalgam* filling.

*Dr. Louis A. Reading.*—The gentleman who preceded me spoke favorably of court-plaster as a lining for cavities, and I will say for the benefit of the young men who heard him, that when I was in college I tried court-plaster on the recommendation of a gentleman who forgot to say what kind of court-plaster we should use, and I made a bad mess of it. I will enlighten you that you may not do as I did. Having heard something said about its use, I tried it by putting a piece of black court-plaster under the proximal surface of a superior lateral incisor. About six months after that, when the patient came back, I noticed that the tooth was terribly discolored. I had bought some gold from a student, and I thought it probable that I had used an inferior article, so I removed the filling and called the attention of the demonstrator to it, who immediately said, "You have used dark court-plaster." I said, "Of course I have. The gentleman told us to use court-plaster, but he did not tell us what kind." Now, if any of you employ court-plaster in a tooth, please use white court-plaster, and not dark.

*Dr. Hayhurst.*—It is always pleasant to me to have the compliments of my friends, and I feel grateful for their commendations now; but the discussion has, it seems to me, got a little twisted out of its legitimate direction, and the point I wished to make in the essay is nearly lost sight of. I would be very sorry indeed, to ignore the philosophy and spiritualism and metaphysics that have made their appearance; I do not object to them, but I want to call your attention to the point I desired to make. My idea in writing that essay was, not to advocate gold over any other material, but simply to call your attention to the manner in which gold foil will work. I do not wish to say anything against amalgam, and did not desire to go into the philosophy of decay; I thought I ignored that, except so far as it was an evil influence that was to be kept out, and in doing so I indicated the proper method of keeping it out and the proper substance to use. Gold foil has been, and I don't know but it is yet, supposed to be the best material manufactured for filling that would accomplish the purpose for which the operator was working, which is to make a tight plug. In considering it I looked a little at the philosophy of the matter, at the method in which that gold filling is made, and the fact that a plug made of foil is either laminated or convoluted in structure, that it is not a homogeneous mass, that it is subject to expansion and contraction, and that it will expand and contract in the direction of the fibres of which it is made. An amalgam filling, or a filling made of solid plastic gold becomes a homogeneous mass, without a laminate structure, and the expansion and contraction will generally be made in a rigid way that forces it against the sides of the tooth, which must yield, and then there is nothing to make that contract again, and so, when the filling shrinks it leaves a space. Now I ask why we reject plastic gold? Is it not on account of the fact that it becomes a homogeneous mass, and expands in the manner and with the result I have described? There is nothing in it to give; when expansion takes place, it pushes the sides of the tooth apart, and when it contracts a cavity is left around it, through which the fluids of the mouth penetrate. Amalgam acts in the same way. Possibly some of the plastic fillings may adhere to the tooth-walls; the phosphates may do so, and if they do they make a better filling in this respect than amalgam.

*Dr. Osmun.*—Will Dr. Hayhurst explain how a filling can expand in the direction of the laminæ of the tooth, if it is made solid, more than in any other direction?

*Dr. Hayhurst.*—I alluded to the laminate structure of the filling, not of the tooth.

*Dr. Rhein.*—Dr. Hayhurst has taken entirely wrong ground on this question. There is, of course, some contraction and some expansion in all metals, but the amount of such contraction and expansion in a filling is infinitesimal, and it has no appreciable effect upon the tooth, nor upon the cavity of the tooth which it restores to its former condition. He asks why plastic gold has fallen into disuse. Because it cannot be impacted solidly, and because of the impossibility of getting a sufficient amount of gold into the cavity. It is for this reason that I believe in impacting the gold with the aid of the electro-magnetic mallet, because you can get more gold into the cavity, and it can be inserted in the cavity in a perfectly homogeneous and solid plug. There is no truth in the assertion that perfect gold fillings come back leaking. The filling that will bear the closest scrutiny under the magnifying glass, and defy the detection of anything imperfect at the margin of the gold and enamel, will always remain perfect unless natural causes produce fresh destruction of the tooth substance. If the exterior of the cavity is once hermetically sealed it will never leak.

*Dr. Wallace.*—Dr. Bodecker spoke of lining cavities with oxy-phosphate and oxy-chloride of zinc, and I would like to ask if he allows that lining to come up to the marginal surface. I believe there are no plastic fillings that are considered insoluble; I have never found any yet; and that being the case, the phosphates would be more readily acted upon by the fluids of the mouth than gold would be, and there would be chance for decay at the marginal surface.

*Dr. Bodecker.*—I have used oxy-chloride of zinc under gold and amalgam fillings for eight or ten years, in all cavities where I was able to, and I do not think I have seen half a dozen failures. It ought by no means to extend to the margin of the cavity, for it will surely be dissolved there. By filling up the greater bulk of a large cavity with oxy-chloride or oxy-phosphate to a certain extent you overcome the thermal changes, and also decrease the quantity of gold or amalgam, so that very little contraction or expansion is possible. If I have a very large contour in a molar or a bicuspid to restore. I would never think of building it up altogether with gold; if possible I would use oxy-phosphate of zinc for the bulk of the filling, except it were a central or lateral incisor in which there was not room for it. But in any cavity where a cement filling is admissible it should be used, and the edges of the cavity ought always to be very smooth and overlapped either by gold or amalgam, the cement never being allowed to reach quite up to the edge of the dentine. You must always have sufficient anchorage for your filling material in the dentine, except in the buccal and lingual walls of badly decayed bicuspids and molars. In this class of cavities I regard it sufficient to make one or two starting-points in the cervical portion, and another good anchorage in the grinding surface of the tooth, which will perfectly secure the filling even if there is no other anchorage. But always be careful to let the gold overlap the enamel. It is better to let the edges of a gold filling be seen than to leave the margin of the cavity imperfectly protected, or fractured during the introduction of the gold.

With reference to the use of gutta-percha under gold fillings, I would like to say that I never use gutta-percha in a thick layer, but always in a chloroform solution. In different cases I use a different strength. If I want to line a cavity with oxy-chloride or oxy-phosphate I use a pretty thick solution of gutta-percha previous to the introduction of the cement, because I regard it unsafe to place either an oxy-phosphate or oxy-chloride filling in the vicinity of the pulp, directly upon dentine. In superficial cavities, however, which are to be filled with gold or amalgam directly, the gutta-percha solution must be quite thin.

*Dr. Wallace.*—I have used plastic fillings to some extent, but I have had better success with gold than with amalgam. I have seen two fillings, one gold and one amalgam, both put in with the most careful manipulation, subjected to a microscopical test, and the gold filling proved to be tight while the amalgam filling was not. I have found on examining a number of fillings under the microscope, that the gold fillings were really more perfect than the amalgam.

*Dr. Watkins.*—The filling materials seem to be mixed this evening, especially gold and amalgam. A few years ago we were taught that we must not allow gold and amalgam to come in contact. Are we turning the dogs into the sheep pen? I have been practicing a method of partly filling cavities with amalgam and then finishing with gold, especially in the proximal surfaces of the bicuspids and molars. I place a piece of thin platinum plate around the tooth as near as possible to its shape and contour. Bind it there with silk floss, and then mix some amalgam as dry as possible, place that in the bottom of the cavity and pack it very hard, putting in considerable more than I want so as to work all the mercury to the top. Then remove a portion of that, cutting down to the hard, solid amalgam where there is no excess of mercury, and I then place the gold on that. The few first pieces are embedded in the surface of the amalgam, and the gold is built upon them. In that way I am positive that the cervical walls of the cavity are perfect and the lower part of the cavity entirely filled, whereas in filling with gold only we cannot always be positive. There are places we cannot see to fill as they should be filled. We do not know exactly where we put our gold. I think we can save teeth by this mode of filling that would not be saved by the use of gold only.

*Dr. J. Allen Osmun*, of Newark, read a paper on the legal and moral responsibility of dentists in the administration of nitrous oxide gas.

Dr. Osmun's paper was published in the August number of THE INDEPENDENT PRACTITIONER.—EDITOR.

*Dr. Stockton.*—Without attempting to discuss the paper, I want to say that I would be very glad if Dr. Osmun could be induced to continue the subject, with special reference to the effects of anæsthesia upon the human system, and I offer a motion that he be requested to do so in another paper.

Dr. Stockton's motion was carried.

*Dr. Atkinson.*—Sometimes circumstances place you in a position where you are supposed to know more than you do know, and if you have the modesty to say so, and confess your ignorance, you will be likely to be esteemed below your merit.

I have no word of anything but commendation for the paper, and the manner in which the subject is treated. I think it argues well for Dr. Stockton to push his mind forward and say there was something more in the same mine whence this was digged, that would lead to something like a scientific apprehension of what we are about when we deprive our patients of consciousness. I do not propose to occupy your time in giving a review of the paper, which is evidently the product of much thought, reading, care, and a keen mental appreciation of the subject.

*Dr. Hayhurst.*—I know something about the legal points that have been stated, and I know that there is a delicacy in exactly separating what may be termed criminal action from that which is not criminal. We are called upon under almost every circumstance that we can imagine to administer this direful agent, and from dentists all over the land we hear that there is no danger in giving nitrous oxide, and patients, who say: "Doctor, I am perfectly satisfied to take the gas; I have done it so frequently that it is not necessary to go through an examination to find out whether

I am a fit subject or not;" and so you may be induced to chance it. According to the decisions given in the various cases recited in the paper you see how careful you must be to navigate safely between these several points in order to secure yourself against legal prosecution. You have not only your own judgment to insure against fallibility, but your patient is often so anxious to take the gas in order to have a tooth extracted without pain, that he or she will tell you that which is not true in regard to their state of health, and you are often misled. I remember a case where I carefully administered ether to extract some teeth, and it was a successful operation, so the lady told me. Before the administration I questioned the lady, and asked her whether she had consulted her family physician in the matter, and whether she had been under treatment for any disease which might be inimical to ether. She told me she had the concurrence of her physician, and that she had never been under treatment for anything that would interfere. A few days afterwards I heard from an intimate friend of hers that she went home and told, "How I fooled that dentist; you know the doctor has been attending me for heart disease." I said to the lady, "Yes, she imposed upon me, but she did not injure me as much as she might have injured herself by her falsehoods, because her life was in her own hands; she placed it in mine with a lie, and deserved to suffer for it." There are dentists who do little else but administer this agent, and say there is no danger in it, but by the examples given us you will find that it is a very delicate matter. How often have I looked upon the face of a patient as she lay back in the chair, her eyes closed, her countenance pallid, and the marks of death upon her lips, so that any person in the room would suppose that her soul had taken its flight to the regions of eternity; and yet it was not death, but so near it that one may see how nice an operation this is, and how near we tread upon dangerous ground. I call upon all members of the profession to deliberate, and use all the precautions and the very best judgment they have concerning these agents, instead of making wholesale statements that there is no danger in anæsthesia, not because they desire to escape the grappling-irons of the law, but in order that they may be morally blameless in their operations. If you do the very best you know how it will not be too good. Nine-tenths of the people who require our services cannot expect to have the highest attainments in dentistry at their beck and call. They must go to New York, Buffalo, Philadelphia, and other great centres of wealth and population in order to get the very best knowledge, but all of you should use to the fullest extent the talents God has given you in endeavoring to preserve the lives and promote the health of the patients who come into your hands.

*Dr. Louis A. Reading.*—I have had considerable experience in the administration of ether, much more than with nitrous oxide gas. I was called upon a few weeks ago to administer ether to a lady who was to undergo an operation for cancer. The physician in charge had neglected to advise his patient in regard to eating, and she had just taken a hearty meal before we arrived; the operation was to take place at two o'clock, and she had dined at one. The only part I had in the operation was to administer the ether. I proceeded with it quite satisfactorily, but when I was through with the stage of excitement I noticed that she began to be a little nauseated. I discontinued the ether a moment to give her a chance to vomit her dinner. A piece of beefsteak that she was about to throw off got lodged in the æsophagus, and, although it did not stop her respiration I noticed that she was considerably embarrassed, and I called the doctor's attention to it. I got a pair of forceps and grasped her tongue while he removed the piece of beefsteak. I simply relate this case to show the importance of taking precautions before we administer an anæsthetic, particularly ether, and to compliment the Doctor on his remarks upon that point. We all know how important it is to caution our patients not to eat heartily just before taking an anæsthetic.

A motion to pass the subject was carried.

Dr. E. H. Bunting, Senr., read a paper entitled Æsthetic Dentistry. (See page 460.)

*Dr. Hayhurst*—What is æsthetic dentistry? What is beauty? We may say one thing is beautiful or æsthetic, and another person will say another and very different thing is beautiful. The inhabitants of certain districts of Asia color their teeth black. Their highest type of beauty would be a set of black teeth ; but that would not pass for æstheticism here. We like white, pearly teeth, perfect and regular, that give a pleasant expression to the countenance, and no marring of nature's mouth-piece shall take place under this idea of æsthetic dentistry. We must go farther back than the treatment the dentist gives; we must go back to pure lives and simple habits; back beyond our immediate ancestry, whose "eating of sour grapes caused their children's teeth to be set on edge"; we must go back to that which produces this characteristic of the American face, the narrow and compressed jaw. As black teeth are now considered beautiful by some, a narrow jaw, and some things that are now abhorrent may some day be esteemed beautiful; therefore I ask whether there is any standard of beauty except such as is made by habit and education?

*Dr. J. A. Robinson.*—I beg to say a word with regard to the meaning of æsthetic. I should define it as that which is perfectly harmonious; that which corresponds to our highest type of beauty in the civilization of to-day. And while I have been pained at seeing some of the deformities that have been brought about by modern dentistry in the application of artificial dentures, I would say that I believe æsthetic dentistry means that which restores the contour of the face to its normal condition and makes the features of the person as presentable as possible to their neighbors and to the world. I have just come from Boston, and while there I called on a friend whom I had not seen for a few years, and I was struck with her altered appearance. I was almost horrified, for by the application of an artificial set of teeth her face had been so changed and distorted that I almost shrunk from contact and salutation with the woman who had once seemed to me so different. "I said, "what have you done to yourself? Age could not have made such a change in a face that was once beautiful! You have had a new set of artificial teeth." I took them out of her mouth, and with a small spirit lamp, a pen-knife and some modeling composition, I molded them over in my hands, attaching to the outside sufficient material to restore the face to its natural fullness and contour, and when I

presented the lady to her daughter and grandchildren they hardly knew her, her appearance was so changed and improved. Therefore, I say that æsthetic dentistry is that kind of dentistry that restores the features, not with pearly white teeth, as has been said here, but with teeth that correspond to the features, and the application of plumpers, if you please, replacing by artificial substitutes what has been lost, so that the person may, as nearly as possible, represent what she did when the natural teeth were in their proper place, and nature had not been distorted by the use of the things that are sometimes worn, and which are very offensive to those who have the least idea of harmony in the human countenance.

(Concluded in October Number.)

